# New insights regarding origin of monosomy occurrence in early developing embryos as demonstrated in preimplantation genetic testing

**DOI:** 10.1186/s13039-022-00582-5

**Published:** 2022-03-21

**Authors:** N. Samara, S. Peleg, T. Frumkin, V. Gold, H. Amir, Einat Haikin Herzberger, A. Reches, Y. Kalma, Dalit Ben Yosef, F. Azem, M. Malcov

**Affiliations:** 1 IVF Lab and Wolfe PGD-Stem Cell Lab, Fertility Institute, Tel-Aviv Sourasky Medical Center, Weismann 6, Tel Aviv, Israel; 2 Department of Cell Biology and Development Biology, Sackler Faculty of Medicine, Tel-Aviv University, Tel Aviv, Israel

**Keywords:** Early embryo aneuploidy, Haplotype, Monosomy, Paternal origin, Polymorphic markers, Preimplantation genetic testing

## Abstract

**Introduction:**

Analyses of miscarriage products indicate that the majority of aneuploidies in early developing embryos derive from errors occurring during maternal meiosis and the paternal contribution is less than 10%. Our aim was to assess the aneuploidy (mainly monosmies) frequencies at the earliest stages of embryo development, 3 days following fertilization during In vitro fertilization (IVF) treatments and to elucidate their parental origin. Later, we compared monosomies rates of day 3 to those of day 5 as demonstrated from Preimplantation Genetic Testing for Structural chromosomal Rearrangement (PGT-SR) results.

**Methods:**

For a retrospective study, we collected data of 210 Preimplantation Genetic Testing for Monogenic Disorder (PGT-M) cycles performed between years 2008 and 2019.This study includes 2083 embryos, of 113 couples. It also included 432 embryos from 90 PGT-SR cycles of other 45 patients, carriers of balanced translocations. Defining the parental origin of aneuploidy in cleavage stage embryos was based on haplotypes analysis of at least six informative markers flanking the analyzed gene. For comprehensive chromosomal screening (CCS), chromosomal microarray (CMA) and next generation sequencing (NGS) was used.

**Results:**

We inspected haplotype data of 40 genomic regions, flanking analyzed genes located on 9 different chromosomes.151 (7.2%) embryos presented numerical alterations in the tested chromosomes. We found similar paternal and maternal contribution to monosomy at cleavage stage. We demonstrated paternal origin in 51.5% of the monosomy, and maternal origin in 48.5% of the monosomies cases.

**Conclusion:**

In our study, we found equal parental contribution to monosomies in cleavage-stage embryos. Comparison to CCS analyses of PGT-SR patients revealed a lower rate of monosomy per chromosome in embryos at day 5 of development. This is in contrast to the maternal dominancy described in studies of early miscarriage. Mitotic errors and paternal involvement in chemical pregnancies and IVF failure should be re-evaluated. Our results show monosomies are relatively common and may play a role in early development of ART embryos.

**Supplementary Information:**

The online version contains supplementary material available at 10.1186/s13039-022-00582-5.

## Introduction

Human embryos present a strong tendency to aneuploidy occurrence because of meiotic and mitotic errors during diverse developmental stages. Previous studies demonstrate that up to 35% of clinical pregnancies are diagnosed with aneuploidy [1]. Additionally, the relatively low rate of live births achieved from in vitro fertilization (IVF) cycles compared to the number of transferred embryos is mainly attributed to aneuploidy [2]. When early developing embryos are chromosomally investigated, a higher percentage of aneuploidy is observed as compared to later embryonic stages, probably due to natural selection and random correction processes [3]. Analyses of the gestational products in early miscarriages indicate aneuploidy mainly of maternal origin [4].This may be explained by the complexity of meiosis mechanism in oogenesis and its extremely prolonged arrest of meiosis that is prone to errors, as compared to spermatogenesis [5]. It is well established that meiotic errors resulting in embryo aneuploidy correlate with maternal age, exceeding 40% of pregnancies at age > 40 years, and each chromosome has different susceptibility to aneuploidy [6]. However, post-meiotic abnormalities that lead to mosaicism, polyploidy and haploidy, are not related to advanced maternal age [7]. Sperm aneuploidy also increases with paternal age [7, 8], yet, according to previous studies aneuploidy affects less than 10% of sperm cells [9, 10].The genomic constitution of single sperm cells was recently analyzed suggesting a significant contribution of the paternal gamete to embryo aneuploidy [11, 12]. Moreover, a relatively high sperm disomy was correlated with increased aneuploidy in preimplantation genetic testing for aneuploidy (PGT-A) diagnosed embryos [13].

Diagnosis of embryo aneuploidy, as well as other genetic alterations, was enabled by introducing of PGT as was first reported by Handyside et al. in 1990 [14]. PGT was initially designed for the diagnosis of monogenic disorders and structural chromosomal rearrangement, and has become a common practice for the identification of various familial disorders [15, 16]. Since then molecular genetic technics had greatly improved, increasing the accuracy and reliability of PGT-M, mainly due to the implementation of haplotype analyses. Haplotype is a group of DNA loci localized close on the same chromosome. Due to the high proximity of loci, crossing-over events are infrequent among them thus they are inherited together from a single parent's allele. This feature allows the parental identification of the analyzed allele. For characterization of haplotypes, analyses of Short Tandem Repeats (STR) are performed [17–19].

PGT-M based on haplotype characterization permits not only a highly accurate discrimination between normal and mutated parental alleles [20], but also make it possible to identify chromosomal gain or loss and to determine the parental origin of aneuploidy [21–24].

The increasing genomic data alongside with advanced single cell molecular techniques, led us to challenge the notion of maternal dominance and parental contribution on early development embryonic aneuploidies.

## Materials and methods

### Study population

This retrospective study included two groups pf patients. First group included 113 patients, carriers of monogenic Mendelian mutations who underwent 210 PGT-M cycles. Second group included 45 patients, carriers of chromosomal structural rearrangement, who underwent 90 PGT-SR cycles. All patients in this study underwent PGT-M or PGT-SR in our unit between the years 2008 and 2019.

### Establishments of PGT analyses systems

Prior to PGT-M treatment, the familial mutation was validated in genomic DNA samples isolated from peripheral blood of the carrier patients and other family members (parents, affected family member, or from a previously aborted foetus). For accurate discrimination between the mutated and normal alleles, detailed informative haplotypes were defined by at least six informative polymorphic informative markers as retrieved from genomic databases, and were most based on short CA tandem repeats (STR). Those informative markers flanked the familial mutation and were located up to 1.5 Mb upstream and downstream of the causative mutation site [18, 23, 24]. Out of a wide panel of STRs for each genotype determination, the most informative ones were chosen for each patient who underwent PGT-M. Prior to the PGT-M cycle, single leukocytes were isolated from carrier patients, parents, or other affected family members and subjected to single-cell multiplex-nested PCR, that simultaneously amplified the familial mutation and flanking STRs. This is a pivotal test performed for the assessment of the feasibility and reliability of diagnosis [17, 18, 25]. High amplification rates (> 95%), low allele dropout rates (ADO) (< 15% for each locus) and the total absence of false positive or negative results in the analysis of all single leukocytes allow the onset of the PGT-M cycle.

In PGT-SR, comprehensive chromosomal screening was performed according to manufacturer instructions, and there was no need for personal adaptations and establishment of a unique diagnosis system except for validation of the reported chromosomal rearrangement by FISH analysis, prior to PGT-SR.

### Ovarian stimulation, fertilization and embryo culture and biopsy

IVF PGT-M/SR cycles were initiated with ovarian stimulation, and oocytes were retrieved 34–36 h following the triggering of ovulation. The oocytes were denuded of cumulus cells, and fertilization was performed by intracytoplasmic sperm injection (ICSI). Fertilized oocytes were examined 18–20 h after ICSI for the presence of pronuclei. All embryos were incubated in an integrated EmbryoScope (Unisense FertiliTech A/S, Århus, Denmark) time-lapse monitoring system from the time of fertilization until blastocyst stage of embryonic development. Embryo morphology and developmental events were recorded, and cleavage-stage embryos were assessed prior to biopsy [26].

For embryo biopsy in PGT-M, the zona pellucida (ZP) was perforated using an in-contact laser, and a single blastomere was aspirated from day 3 embryos that were composed of at least six cells (blastomeres). The embryo biopsy for PGT-SR began with ZP slighting at day 3 using a laser beam, followed by gentle aspiration and laser slicing of the expanded blastocyte trophectoderm's hernia, at day 5–6. This trophectoderm sample, usually includes 5–10 cells. Both blastomere and trophectoderm embryonic samples were washed and placed in PCR tubes under sterile conditions and were then exposed to high temperatures for DNase inactivation. They were then kept at − 20 °C until analyzed [17].

### PGT procedure

In the PGT-M cycle, we used a calibrated tailor-made diagnosis system for each family as aforementioned. During PGT-M, single blastomeres and control leukocytes were lysed in order to expose DNA to the first PCR ingredients and subjected to parallel amplification of both mutation and selected panel of polymorphic markers. Single leukocytes with known genotypes served as positive and negative controls for estimating the accuracy and reliability of the analysis as described previously by Malcov et al. [18]. Aliquots of the first PCR reaction were subjected to second amplification using nested PCR primers. For the discrimination between normal and mutated alleles, amplified informative markers, expansion mutations and indel mutations were analyzed using a fragment analyser (3130xl genetic analyser, Life Technologies, Carlsbad, CA). Other point mutations were tested by enzymatic restriction followed agarose gel separation.

For PGT-SR, trophectoderm cells were subjected to whole-genome amplification (WGA) (PicoPLEX WGA kit, Rubicon Genomics, Ann Arbor, MI). The uniformly amplified embryonic DNA allowed a reliable diagnosis when microarray analysis or NGS (VeriSeq PGS kit-Illumina) was performed [27]. For microarray analysis, WGA samples of trophectoderm and control cells were differently labelled with fluorescent colours, co-hybridized to a DNA array and washed following overnight hybridization (GenetiSure Array Kit and Sue Tag DNA Labeling Kit Agilent Technologies, Texas,USA).Slides were screened, and software compared each sample to a reference control (Agilent CytoGenomics software).The comparison between fluorescence of embryo samples and normal control samples allowed for the determination of whole chromosomal constitution and enabled ploidy characterization of each tested embryo.

### Data collection

We summarized data obtained from haplotype analyses of 2083 day 3 embryos from the PGT-M cycles and compared the results to CCS results of 432 day 5 embryos from the PGT-SR cycles.

### Analyses and comprehension of results

During PGT-M, normal and mutated alleles were determined by the use of highly detailed informative haplotypes. Any absence or addition of a specific allele was also defined, and the parental origin of gain or loss was elucidated. Monosomy was determined when the embryonic genotype presented only one parental haplotype (maternal or paternal) in all informative tested markers. Trisomy was determined when one of the parents transmitted two different alleles and three different haplotypes could be demonstrated in the analyzed embryo. This chromosomal status refers to two-parent homolog (BPH) aneuploidy, and it is clearly demonstrated by haplotype analysis. However, another type of trisomy presented by single-parent homolog (SPH), where two identical chromosomes originate from one parent, is misdiagnosed by haplotype analysis [28]. Therefore, we deliberately did not compare trisomy rates at day 3 to day 5, and our study concentrated mainly on monosomy rates in day 3 embryos and compared them to monosomy in day 5 embryos.

Alterations in 9 selected chromosomes, as demonstrated by haplotype analyses, were compared to CCS of PGT-SR results that demonstrated also aneuploidies in chromosomes that are not a part of the familial translocation. The comparison of aneuploidy rate at day 3 to day 5 embryos was based on the assumption that in cleavage stage embryos trisomies and monosomies are significantly more common than segmental chromosomal deletions or duplications in a certain analysed DNA locus [29].

## Results

We reviewed and analyzed haplotype data of 2083 single blastomeres biopsied from cleavage-stage embryos obtained from 210 PGT-M cycles of 113 couples. In Table 1, we present data of PGT-M cycles analyzing 40 genes located on chromosomes 1, 2, 6, 7, 16, 17, 19, 20 and chromosome X. Chromosomes were selected and reviewed randomly within the study’s time frame. The mean age for maternal patients ranged from 29.5 to 34.5 years (mean 32.5) and the age of the paternal patients ranged between 32.7 to 39.6 years (mean 35.4 yrs.). In our study, as might be expected, the longest chromosome, chromosome 1, represented eight monogenic disorders, the highest number among the chromosomes. However, the highest numbers of embryos 723 were analyzed for mutations on chromosome 7, perhaps due to the prevalence of cystic fibrosis mutations that are routinely screened in our population, in Table 2 we demonstrate the following details: the nature and mode of inheritance of the tested mutation, the carrier status (de-novo or inherited) and parental origin.

**Table 1:** Characteristics of the patients who underwent PGT-M cycles according to tested chromosome in day 3 embryos

Tested chromosome	No. of patients	Age-years (mean) Maternal	Age-years (mean) Paternal	No. of cycles	No. of embryos	No. of analyzed genes
1	17	34	35.5	55	316	8
2	8	36	39.6	8	118	6
6	11	29.5	32.7	7	202	5
7	31	33	36.3	75	723	5
16	11	30.5	32.8	9	151	4
17	9	30.5	34.7	17	132	3
19	18	33	35.5	18	331	5
20	2	34.5	34.9	9	26	1
X	6	32.5	33.7	12	84	3
Total	113	32.5	35.4	210	2083	40

**Table 2:** Nature and mode of inheritance of the tested mutation, the carrier status (de-novo or inherited) and parental origin per tested chromosome

Chromosome tested	Recessive mutation	Dominant mutation	X-linked mutation	Paternal Mutation	Maternal mutation	De-novo
1	8	9	–	11	14	–
2	2	6	–	3	7	–
6	9	2	–	8	9	2
7	26	5	–	27	30	–
16	1	10	–	4	8	–
17	2	7	–	5	6	–
19	2	16	–	2	18	–
20	0	2	–	1	1	–
X	–	–	6	0	6	–
Total	50	57	6	61	99	2

The haplotype analyses revealed a numerical alteration of the specific tested chromosome in 7.2% (151) of the tested embryos. Of all alterations that could be found,47.1% (71) were of paternal origin, and 52.9% (81) were of maternal origin (Table 3). When alterations were classified, 87.5% (133) displayed monosomy, and only 12.5% (19) presented trisomy (Table 4). However, as we mentioned earlier, due to methodology limitations, we detected only a minority of trisomy proportions.

**Table 3:** Parental origin of chromosomal aneuploidy in day 3 embryos according to haplotype analysis, in PGT-M cycles

Chromosome tested	No. of embryos	No. of embryos with aberration	Maternal origin	Paternal origin
1	316	29 (9.1%)	16 (55.2%)	13 (44.8%)
2	118	10 (8.4%)	2 (20.0%)	8 (80.0%)
6	202	4 (1.9%)	1 (25.0%)	3 (75.0%)
7	723	51 (7.0%)	32 (62.7%)	19 (37.2%)
16	151	17 (11.2%)	10 (58.8%)	7 (41.2%)
17	132	6 (4.5%)	5 (83.3%)	1 (16.7%)
19	331	19 (5.7%)	8 (42.1%)	11 (57.9%)
20	26	6 (2.3%)	4 (66.7%)	2 (33.3%)
X	84	9 (10.7%)	2 (22.2%)	7 (77.8%)
Total	2083	151 (7.2%)	80 (52.9%)	71 (47.1%)

**Table 4:** Parental origin of monosomy and trisomy at day 3 embryos according to haplotype analysis, in PGT-M cycles

Chromosome tested	No. of embryos with aberrations	Monosomy	Trisomy	Maternal monosomy	Paternal monosomy	Maternal trisomy	Paternal trisomy
1	29 (9.1%)	26 (89.7%)	3 (10.3%)	13	13	3	0
2	10 (8.4%)	9 (90.0%)	1 (10.0%)	2	7	0	1
6	4 (1.9%)	4 (100%)	0	1	3	0	0
7	51 (7.0%)	44 (86.3%)	7 (13.7%)	24	20	7	0
16	17 (11.2%)	15 (88.2%)	2 (21.2%)	8	7	2	0
17	6 (4.5%)	6 (100%)	0	5	1	0	0
19	19 (5.7%)	14 (73.7%)	5 (26.3%)	6	8	2	3
20	6 (2.3%)	5 (83.3%)	1 (16.7%)	3	2	1	0
X	9 (10.7%)	9 (100%)	0	2	7	0	0
Total chromosomes (9)	151 (7.2%)	132 (87.5%)	19 (12.5%)	64/132 (48.5%)	68/132 (51.5%)	15/19 (78.9%)	4/19 (21.1%)

It can be seen in Table 4 that day 3 embryos demonstrated almost equal maternal and paternal contributions to monosomies, at 48.5% and 51.5%, respectively, while with trisomies, maternal origin made up 78.9% versus 21.1% for those of paternal origin.

Monosomy rates observed in day 3 were compared to aneuploidy data for day 5 embryos. For this purpose, we collected PGT-SR data on 432 embryos analyzed for a known familial structural rearrangement using a comparative genomic hybridization (CGH) array. Comprehensive chromosomal screening for 432 embryos resulted that 6.2% (27) of the embryos failed to be diagnosed due to the low quality of the biopsy sample’s WGA. Aneuploidy was demonstrated in 30.1% (122) of the samples, out of which 64.8% (79) presented single chromosomal gain or loss, while the other 35.2% of embryos (43) presented multiple alterations (Table 5). Analyzing aneuploidy in all chromosomes at day 5 revealed that day 5 trophectoderm present slightly higher rate of trisomy compared to monosomy, single trisomy 58.2% (46) versus 41.8% (33) single monosomy, as shown in Table 5. When monosomy on day 3 was compared to the same chromosome on day 5, a decrease in monosomy rate was detected in all analyzed chromosomes (Table 6).

**Table 5:** Chromosomal aneuploidy in day 5 embryos according to CCS analysis during PGT-SR

No. of patients	45
Maternal age (mean)	34.4
Paternal age (mean)	36.5
No of patients for PGT-SR	45
No of cycles	90
No. of examined embryos	432
No. of embryos with no result (%)	27/432 (6.2)
No. of embryos with result (%)	405/432 (93.8)
No. of euploid embryos (%)	283/405 (69.9)
No. of aneuploid embryos (%)	122/405 (30.1)
No. of embryos with multiple chromosomal aberrations (%)	43/122 (35.2)
Total aberrations (dispersed throughout 43 embryos)	194
Chromosome monosomies (%)	78/194 (40.2)
Chromosome trisomies (%)	116/194 (59.8)
No. of embryos with single chromosomal aberration (%)	79/122 (64.8)
Single chromosome monosomy (%)	33/79 (41.8)
Single chromosome trisomy (%)	46/79 (58.2)

**Table 6:** Monosomy rates of day 3 embryos compared to monosomy rates of day 5 embryos

Tested chromosome	Chromosome size in bp	Day 3 rate of monosomy *	Day 5 embryo rate of monosomy**	Day 5 monosomy rate/Day 3 monosomy rate
1	249,250,621	26/316 (8.2%)	3/405 (0.7%)	0.09
2	243,199,373	9/118 (7.6%)	6/405 (1.5%)	0.19
6	171,115,067	4/202 (2.0%)	5/405 (1.2%)	0.62
7	159,138,663	44/723 (6.0%)	6/405 (1.5%)	0.24
16	90,354,753	15/151 (9.9%)	22/405 (5.4%)	0.55
17	81,195,210	6/132 (4.5%)	0	–
19	59,128,983	14/331 (4.2%)	2/405 (0.49%)	0.12
20	63,025,520	5/26 (19.2%)	7/405 (1.7%)	0.09
X	155,270,560	9/84 (10.7%)	4/405 (1.0%)	0.09

^*^Rate of monosomy calculated by number of embryos with monosomy of the tested chromosome divided by total number of embryos tested by the specific chromosome

^**^Rate of monosomy calculated by number of embryos with monosomy (single and multiple) of the specific chromosome divided by number of tested embryos with results (405), as all embryos were tested for all chromosomes by CCS

## Discussion

The aim of the present study was to characterize the nature of aneuploidy mainly monosomy in early stages of embryo development and its parental origin, mainly by the use of haplotype analysis during PGT. A comparison of day 3 aneuploidy to CCS of day 5 embryos, as demonstrated by PGT-SR, revealed a remarkable reduction of chromosomal monosomies with embryo development. McCoy et al. demonstrated evidence of selection against complex aneuploidy occurring early, in the postfertilization period before day 5 [30].

In this study, we contribute to the understanding of common early cytogenetic events of chromosomal gain or loss mainly caused by random mitotic errors and resulting in mosaicism and embryonic aneuploidy. Here we suggest possible mechanisms explaining developmental aspects and discrepancies concerning significant paternal contribution to monosomy and trisomy throughout embryo development.

Early embryo aneuploidy is the common explanation for poor development and implantation failure [31]. In our study, we analyzed 2083 day 3 embryos of PGT-M patients with maternal average age of 32.5 years, and paternal average age of 35.4 years. In overall, we found an average rate of 7.2% events of chromosomal alteration when a single chromosome was analyzed each time. Analyzing haplotypes of nine selected chromosomes, we found different chromosomal tendency to aneuploidy, with chromosome 6 presenting exceptionally low rates of abnormalities and higher rates were presented by chromosome 16 and chromosome X. A similar divergence of aneuploidy between chromosomes 6 and 16 was reported by Rabinowitz et al. [28] and may be explained by the immunological importance of the HLA region of chromosome 6 and also by the natural inactivation of chromosome X. The highest rate of aneuploidy was presented by chromosome 20, but with a relatively small number of embryos analyzed, and poor ovarian reserve frequently correlated with increased aneuploidy.

The assented concept of embryo aneuploidy, mainly based on analyses of aborted foetuses, attributes chromosomal errors to maternal origin and proved to be age dependent. However, when we investigated parental origin of all aneuploidy errors in cleavage stage embryos, we found that 52.9% were of maternal origin, and 47.1% were of paternal one. Subdivision of aneuploidy into monosomies and trisomies, revealed a similar maternal and paternal contributions to monosomies but with a significantly higher maternal contribution to trisomy (78.9% vs. 21.1%). Rabinowitz et al. studied aneuploidy using SNP analysis in blastomeres and presented similar results [28]. In contrast to the low rates of paternal BPH (both parental homolog) trisomies that originated from a meiotic error, demonstrated in our study, Rabinowitz et al. did not find even one case of paternal BPH trisomies in the analysis of 274 day 3 blastomeres [28]. We suggest that although SNP analysis of all chromosomes provides comprehensive information, it is accompanied by relatively high rates of allele dropouts (ADO) as compared to analysis by single-cell multiplex PCR [32]. The ADO of additional haplotypes may miss a paternal BPH trisomy event.

Similar paternal and maternal contribution to monosomy, as clearly demonstrated by our haplotype analyses, is different from the well-accepted maternal dominance observed in day 5 embryos [33] and first trimester spontaneously aborted embryos. Moreover, in addition to the random mitotic errors, maternal chromosomes are subjected also to meiotic errors [34]. Therefore, we would expect higher rate of monosomies of maternal origin compared to paternal ones at day 3. In our study we found almost equal paternal and maternal contribution to day 3 embryonic monosomy, this observation necessitated biological explanation. The most reasonable explanation is that due to strict cell cycle checkpoints, spermatogenesis indeed results in a very low rate of meiotic aneuploidy. Therefore, the observed paternal chromosomal alterations result mainly from mitotic errors rather than meiotic ones. Similar explanation was suggested by Rabinowitz et al. [28]. Less likely is the possibility that spermatogenesis, similar to oogenesis, results in a remarkable aneuploidy, but paternal aneuploidy is negatively selected due to fertilization impairment of the unbalanced sperm cells, or reduced tolerance of embryonic cells to paternal chromosomal alteration as compared to maternal ones. This intolerance can be attributed to a relatively low methylation on paternal chromosomes and higher allelic expression, at this developmental stage, compared to the maternal ones [35–37]. Finally, there is also a probability that the deletion of around 3 Mb demonstrated by haplotype analyses is actually only segmental aneuploidies and categorized by mistake as whole chromosome monosomy or trisomy. However, segmental aneuploidy incidence at day 3 embryos is relatively low compared to trisomy and monosomy [38]. In addition, the chances of identification a random segmental deletion during characterization of a single gene, are very small.

The relatively high prevalence of total monosomies at cleavage stage prompted the comparison to the quantitative CCS analyses of trophectoderm derived from blastocyst embryos. At day 5, the ratio of trisomies and monosomies was converted, and more trisomies were observed due to not only the ability of CCS to diagnose trisomies originating from the same chromosomes but mainly because the embryo can more easily endure a chromosomal gain than a chromosomal loss [39, 40]. The decrease in monosomies rates as demonstrated in Table 6 is attributed to selection process that might be affected by size of each chromosome and by the function of the genes located on it. However, in our study we found no correlation between chromosomal size and the rate of negative selection against monosomies.

The limitation of haplotype analysis to identify only those trisomies demonstrating different haplotypes, turns to contain a significant advantage, as it permits the essential differentiation between meiotic and mitotic chromosomal errors. Therefore, we suggest that the differences between trisomy rates diagnosed by an additional haplotype at day 3 and CCS results of PGT-SR at day 5, hints at a significant contribution of mitotic errors rather than meiotic ones. The incidence of chromosomal errors in early stages of development and their progressive reduction along embryonal cell divisions, explains why mosaicism is still demonstrated at day 5 and the fact that low rate of mosaicism may result in an euploid viable embryo. This sequence of events might explain the dynamics of aneuploidy in early embryos. This hypothesis should encourage the study of innate and environmental factors, that may impair early embryonic cell division in the IVF laboratory.

## Conclusion

Our detailed molecular analyses of aneuploidy during PGT-M demonstrated similar parental origin of monosomies, perhaps dispelling a prior common conception of prominent maternal contribution. In addition, we emphasized the significant contribution of monosomies, probably originated mainly during mitosis, to total chromosomal alterations in cleavage-stage embryos and may appear as a mosaic chromosomal constitution. Monosomy rates further decrease due to selective reduction during the development into blastocyst embryos, probably in a gene-dependent manner, rather than chromosomal size dependent only (Additional file 1).

## Supplementary Information

**Additional file 1**. Description of the accurate analyzed region in each tested gene

## Data Availability

Not applicable.

## Abbreviations

PGT-M: Preimplantation genetic testing for monogenic disorder
PGT-SR: Preimplantation genetic testing for structural chromosomal rearrangement
IVF: In vitro fertilization
CCS: Comprehensive chromosomal screening
CMA: Chromosomal microarray
NGS: Next generation sequencing
ART: Assisted reproductive technologies
STR: Short tandem repeats
