# Supplementary material for: New insights regarding origin of monosomy occurrence in early developing embryos as demonstrated in preimplantation genetic testing
Source: Mol Cytogenet. 2022 Mar 21;15:11. doi: 10.1186/s13039-022-00582-5 (PMC8935781; doi:10.1186/s13039-022-00582-5)
Supplement: Supplementary file 1 — Additional file 1. Description of the accurate analyzed region in each tested gene [file 13039_2022_582_MOESM1_ESM.docx]

Supplementary material :In this table we give detailed description of the accurate analyzed region in each tested gene.

| **Chromosome** | **Gene** | **Genomic coordinates (GRCh38/hg38)-OMIM** | **Analyzed region** |
| --- | --- | --- | --- |
| 1 | RH | 1:25,272,392-25,330,444 | 24,749,942-27,189,332 |
|  | CPT2 | 1:53,196,823-53,214,196 | 51,948,070-54,925,294 |
|  | ABCA4 | 1:93,992,833-94,121,147 | 93,689,407-95,607,048 |
|  | TAR | 1:145,921,555-145,927,483 | 144,014,370-146,968,166 |
|  | Muc1 | 1:155,185,823-155,192,914 | 153,611,773-156,531,292 |
|  | GBA | 1:155,234,451-155,244,626 | 154,181,903-156,531,292 |
|  | CPVT | 1:237,042,183-237,833,987 | 236,529,519-238,389,765 |
| 2 | MSH2 | 2:47,630,206-47,710,367 | 46,890,569- 47,889,320 |
|  | Nem2 | 2:151,485,333-151,734,475 | 150,940,632-154,412,625 |
|  | CMS | 2:174,747,591-174,764,471 | 174,840,165-176097802 |
|  | BDA1 | 2:219,054,423-219,060,920 | 23014513-26701536 |
|  | PAX3 | 2:222,199,886-222,298,997 | 221,723,776-224,663,899 |
|  | COL4A3 | 2:228,029,281-228179508 | 227717344-228978243 |
| 6 | CAH | 6:32,038,414-32,041,643 | 30884928-32,786,142 |
|  | HLA | 6:32,812,767-32,820,466 | 28,058,635-39,191,771 |
|  | MMA | 6:49,430,359-49,463,297 | 47,210,738-50,888,482 |
|  | ARPKD | 6:51,614,684-52,087,624 | 50710400-52861632 |
|  | MSUD | 6:80,106,609-80,469,087 | 78,535,505-80,462,674 |
| 7 | TWIST | chr7:19,155,091-19,157,295 | 18,771,021-19,377,016 |
|  | CCM2 | chr7:45,067,233-45,116,069 | 44,547,860-46,478,509 |
|  | CFTR | chr7:117,120,017-117,308,718 | 116,407,724-118,042,243 |
|  | LQT | chr7:150,944,961-150,978,321 | 148,448,647-151,038,944 |
|  | MNX1 | chr7:156,994,051-157,009,697 | 156,243,198-157,174,383 |
| 16 | TSC2 | 16:2,047,803-2,089,490 | 876,394-3,379,111 |
|  | PKD1 | 16:2,088,710-2,135,898 | 826,079-2,517,442 |
|  | EARS2 | 16:23,533,334-23,568,696 | 22,937,651-24,618,446 |
|  | SALL1 | 16:51,135,981-51,151,269 | 49,653,961-52,009,704 |
|  | CDH1 | 16:68,737,291-68,835,536 | 67,141,029-69,946,242 |
|  | FANCA | 16:89,803,959-89,883,065 | 88,455,030-89,996,289 |
| 17 | ASPA | 17:3,473,645-3,503,404 | 2,834,463-3,990,918 |
|  | TP53 | 17:7,668,420-7,687,489 | 6,527,824-9,865,506 |
|  | CMT1A | 17:15,229,778-15,265,325 | 14,283,837-15,318,957 |
| 19 | ML4 | 19:7,522,623-7,534,008 | 7,319,767-8,238,080 |
|  | SCA6 | 19:13,206,441-13,506,478 | 11,815,283-13,466,584 |
|  | DMPK | 19:45,769,708-45,782,489 | 45045907-47010923 |
|  | BCKDHA | 19:41,397,817-41,425,001 | 40,112,817-42,797,565 |
| 20 | PRNP | 20:4,686,455-4,701,587 | 3,072,512-5,763,610 |
| x | PHEX | X:22,032,324-22,251,309 | 21,585,826-22,947,570 |
|  | HIGM | X:136,648,157-136,660,389 | 134,096,832-136,236,852 |
|  | FMR1 | X: 146,993,469-147,032,647 | 1 |
